# Preoperative assessment of ovarian tumors using a modified multivariate index assay

**DOI:** 10.1186/s13048-018-0419-7

**Published:** 2018-05-29

**Authors:** Hero A. Abdurrahman, Ariana Kh. Jawad, Shahla K. Alalalf

**Affiliations:** 1 Maternity Teaching Hospital, Kurdistan, Erbil, Iraq; 2 Kurdistan Board for Medical Specialists, Kurdistan, Erbil, Iraq; 3 Department of Obstetrics and Gynecology, Hawler Medical University, College of Medicine, Kurdistan region, Erbil, Iraq

**Keywords:** Multivariate index assay, CA 125, Ovarian, Tumor, Physician’s assessment

## Abstract

**Background:**

Preoperative differentiation between benign and malignant masses can be challenging. The aim of this research was to evaluate the performance of a modified multivariate index assay (MIA) in detecting ovarian cancer and to compare the effectiveness of gynecologist assessment, cancer antigen (CA) 125, and MIA for identifying ovarian masses with high suspicion of malignancy.

**Results:**

This prospective observational study included 150 women with ovarian masses who underwent surgery in the Maternity Teaching Hospital from December 2014 to May 2016. Preoperative estimation of modified MIA, assessment by a gynecologist, and CA 125 level correlated with the surgical histopathology. A modified MIA was implemented because of lack of access to the software typically used. Among 150 enrolled women there were 30 cases of malignancy, including 8 cases (26%) of early-stage ovarian cancer and 22 cases (74%) of late-stage cancer. MIA showed high specificity (96.7%) in detecting cancer and a sensitivity of 70%, with a positive predictive value of 84% and a negative predictive value of 92.8%. No significant differences were detected between the MIA results and the histopathology results (*P* = 0.267). For early-stage ovarian cancer, the sensitivity of MIA was 100% compared with 75% for CA 125 alone.

**Conclusion:**

MIA seems to be effective for evaluation of ovarian tumors with higher specificity and positive predictive value than CA 125 while maintaining high negative predictive value and with only a slightly lower overall sensitivity. For evaluation of early-stage ovarian cancer, MIA showed a much higher sensitivity that markedly outperformed CA 125 alone. This modified MIA strategy may be particularly useful in low resource setting.

## Background

Ovarian cancer is the leading cause of mortality among all gynecologic cancers and the seventh most common cancer in females worldwide [1]. Approximately 10% of women will have some form of surgery for an ovarian mass during their lifetime, and although 20% of borderline ovarian tumors appear as simple cysts on ultrasound many of these tumors may proceed to malignancy later on [2]. Women with ovarian malignancies that are managed by specialized care provided by a gynecologic oncologist or in a specialized hospital have an improved mean survival time, making early diagnosis and rapid management of ovarian malignancy of paramount importance [3]. Radiologic and serum markers are relatively insensitive and as a result preoperative differentiation between benign and malignant ovarian masses is problematic, especially in the differentiation of stage I epithelial ovarian cancer. No single ultrasound finding can differentiate between benign and malignant ovarian masses [2, 4].

Estimation of the risk of malignancy is essential when assessing ovarian masses, and the risk for malignancy has been assessed using 80 different models, including the Royal College of Obstetrician and Gynecologists guidelines. Simple models use discrete cutoff values, such as cancer antigen (CA) 125, pulsatility index, and resistive index [2, 5]. The American College of Obstetricians and Gynecologists and the Society of Obstetricians and Gynecologists of Canada have defined guidelines for the management of premenopausal women with a pelvic mass. These guidelines consider the following features suspicious for ovarian malignancy and warranting referral to a gynecological oncologist: serum CA 125 > 200 units/ml, ascites, evidence of abdominal or distant metastasis, or a first-degree relative with ovarian or breast cancer [5, 6]. However, even in the presence of an ultrasonographically defined mass, serum CA 125 cannot reliably distinguish between a malignant or benign mass [7]. Furthermore, despite efforts to define factors that identify risk of malignancy a previous report showed that 30% of premenopausal women with ovarian cancer would not have been regarded as high risk using these guidelines [6].

As early-stage ovarian cancer carries a much more favorable prognosis than late-stage ovarian cancer there is an urgent need to identify subclinical disease. Serological markers are theoretically an ideal approach, but none of the available markers have 100% specificity and sensitivity [6]. The multivariate index assay (MIA) for ovarian cancer is composed of CA 125, beta 2-microglobulin, apolipoprotein A1 (ApoA1), transferrin, and prealbumin. These biomarkers are not used for screening but for evaluating women with a pelvic mass and can significantly improve the predictability of ovarian cancer so that patients can be referred to a subspecialist with expertise in managing ovarian cancer, thus improving clinical outcome [7]. A previous study showed that the MIA demonstrated higher sensitivity and lower specificity in detecting ovarian malignancies compared with physician assessment and CA 125 [8]. Another report showed that MIA demonstrated higher sensitivity and negative predictive value for ovarian malignancy compared with clinical impression and CA 125 in women with ovarian masses [7]. In our locality, gynecologists and general oncologists (there are no gynecologic oncologists in our locality) depend on clinical assessment, ultrasound, magnetic resonance imaging, and CA 125 level for evaluation of ovarian masses.

We aimed to introduce these biomarkers in our hospital but were unable to access the software needed for the MIA assay. Therefore, we conducted this study to evaluate the use of a modified MIA; specifically, we examined the application of a software-independent method using cut-off dependent risk classification to differentiate malignant from benign ovarian tumors and compared the effectiveness of this novel strategy with physician assessment and CA 125 for identification of ovarian masses with high suspicion of malignancy.

## Methods

A prospective observational study was conducted on 150 women who were admitted for surgical management of ovarian masses in the Maternity Teaching Hospital, Erbil, Kurdistan, Iraq, from December 1 2014 to May 1 2016. The Maternity Teaching hospital is a tertiary referral hospital with 313 beds and the only public tertiary care hospital in Erbil Governorate (population of approximately 1,612,692). The hospital serves as a major referral center for other public and private hospitals within Erbil Governorate and provides emergency obstetrics and gynecology service 24 h a day.

The criteria for inclusion in the study were as follows: female aged 18 years or older, a documented ovarian mass with planned surgical intervention within 3 months of imaging, not referred to an oncologist, and consent to undergo phlebotomy. The exclusion criteria were as follows: age less than 18 years, pregnancy, no planned surgical intervention, declined phlebotomy, or had a malignancy diagnosed in the previous 5 years. Menopause was defined as absence of menses for at least 12 months or age ≥ 50 years when the woman was unsure of her menses. All patients provided written informed consent. All patients were interviewed before the surgical intervention.

Preoperative venous blood (5–7 ml) was collected from each patient and placed in BD plastic vacutainer tubes with clot activators. Samples were transferred to the laboratory and centrifuged, and serum was separated as soon as possible to prevent hemolysis. Serum samples were stored at 2–8 °C for up to 2 days or frozen at −20 °C.

The MVI assay incorporates CA 125, beta 2 microglobulin, transferrin, transthyretin (prealbumin), and apolipoprotein A1. CA 125 was measured using the Elecsys CA 125 II tumor marker system (Fujirebio Diagnostics, Tokyo, Japan). The other four markers were measured on the Minineph system using Minineph kits (Binding Site Group, Birmingham, UK).

The OvaCalc software combines the value of each assay and uses the MVI assay algorithm to generate an ovarian risk malignancy index score that ranges from 0 to 10. A score of ≥5 in premenopausal women or a score of ≥4.4 in postmenopausal women indicates a high probability of malignancy. This software was not available to us due to policy decisions of the manufacturing company. The unavailability of the OvaCalc software forced us to use a modified MIA to assess the validity of these biomarkers in combination for screening ovarian tumors for malignancy. We decided to score the results of the assay as follows: positive cases (indicating a high risk of malignancy) were defined when three out of the five markers were positive, and negative cases (low risk of malignancy) were indicated when ≤2 markers were positive. In the individual assays, positive results were defined as CA 125 and beta 2 microglobulin values higher than normal ranges and apolipoprotein A1, transferrin, and prealbumin values lower than the normal ranges (Table 1).

**Table 1:** Laboratory ranges used for the multivariate index assay

Biomarkers	Ranges	Classifications
CA 125	Premenopausal, 200 IU/L Postmenopausal, 35 IU/L	Ranges above the upper limit regarded positive [11]
^a^β-2 microglobulin	1.22–2.46 g/L	Ranges above the upper limit regarded positive
^a^Transferrin	2.24–4.06 g/L	Level below the lower limit is regarded positive
^a^Apolipoprotein A1	1.24–2.02 g/L	Level below the lower limit is regarded positive
^a^Prealbumin	0.216–0.328 g/L	Level below the lower limit is regarded positive

^a^Ranges obtained from The Binding Site Group kits

For each patient, CA 125 was measured and analyzed as part of the MIA and alone. The CA 125 cutoff values were > 200 U/ml for premenopausal women and > 35 U/ml for postmenopausal women according to the published American College of Obstetrician and Gynecologists referral criteria [9].

Normal ranges for the other four biomarkers were obtained from the leaflet provided with the kits (The Binding Site Group, Birmingham, UK) (Table 1).

This modified MVI assay could be used to screen for malignancy before the operation in our low resource setting.

Before surgery, the enrolling gynecologic surgeon performed preoperative assessment by asking the gynecologists whether they considered the ovarian tumor to be malignant based on available clinical information such as physical examination of the patient, family history of malignancy, results of imaging such as ultrasound, and laboratory tests including CA 125, but not the multivariate index assay. The answers were recorded as yes or no. Gynecologists were allowed to use any algorithm to determine their answer, but were not expected to explain how they reached their prediction. The gynecologists’ opinions were recorded before surgery for all patients. During surgery, FIGO staging was used to surgically stage cases suspicious for cancer and the stage was recorded [10]. After surgery, results of histopathological examination were obtained for each patient from the histopathology department in the hospital laboratory.

## Statistical analysis

Data were analyzed using the Statistical Package for Social Sciences (SPSS, version 22). Chi square test of association was used to compare between proportions. When the expected count of more than 20% of the cells in the table was less than 5 (which was attributed to small sample size in the cells of the table), Fisher’s exact test was used [11]. The expected count of each cell of the table was calculated by multiplying the marginal totals and dividing this value by the grand total. McNemar test (2 × 2) was used when the results of the screening tests such as MIA or CA 125 were compared with a gold standard (the confirmatory test was the histopathological findings) of the same patient, as in Table 2.

**Table 2:** Assessing the accuracy of screening tests

		Histopathology results (gold standard)			*P*
		Positive	Negative		
Screening tests like MIA	Positive	TP	FP	TP + FP	
	Negative	FN	TN	FN + TN	
Total		TP + FN	FP + TN	Grand total	

*P* value, determined by McNemar

*TP* true positive, *TN* true negative, *FP* false positive, *FN* false negative

$$ \mathbf{Sensitivity}=\mathrm{TP}/\left(\mathrm{TP}+\mathrm{FN}\right)\times 100;\mathbf{Specificity}=\mathrm{TN}/\left(\mathrm{FP}+\mathrm{TN}\right)\times 100;\mathbf{Predictive}\ \mathbf{value}\ \mathbf{positive}=\left({\mathrm{PV}}^{+}\right):\mathrm{TP}/\left(\mathrm{TP}+\mathrm{FP}\right)\times 100;\mathbf{Predictive}\ \mathbf{value}\ \mathbf{negative}\ \left({\mathrm{PV}}^{-}\right):\mathrm{TN}/\left(\mathrm{FN}+\mathrm{TN}\right)\times 100;\mathbf{Total}\ \mathbf{agreement}=\left(\mathrm{TP}+\mathrm{TN}\right)/\mathrm{Grand}\ \mathrm{total} $$

Microsoft Excel 2007 was used to plot the pie charts. A *p* value ≤0.05 was considered statistically significant.

## Results

A total of 150 women with an ovarian mass were included in the study. The rate of malignancy was significantly higher among menopausal, grand multiparous, overweight, and obese women (*p* < 0.001) (Table 3).

**Table 3:** Association of histopathological results with variables

		Histopathological results						
		Malignant		Benign		Total		
Variables	Categories	*n*	%	*n*	%	*n*	%	*p*
Menopausal status	Premenopausal	6	5.4	106	94.6	112	100.0	< 0.001
	Postmenopausal	24	63.2	14	36.8	38	100.0	
Parity	Nuliparous	8	16.7	40	83.3	48	100.0	0.001
	Multiparous [1–4]	4	7.4	50	92.6	54	100.0	
	Grandmultiparous (parity ≥5)	18	37.5	30	62.5	48	100.0	
Family history of CA (breast, ovary, colon)	Negative	29	20.4	113	79.6	142	100.0	1*
	Positive	1	12.5	7	87.5	8	100.0	
Combined OCP	Negative	27	19.1	114	80.9	141	100.0	0.384*
	Positive	3	33.3	6	66.7	9	100.0	
Fertility drugs	Negative	28	20.0	112	80.0	140	100.0	1*
	Positive	2	20.0	8	80.0	10	100.0	
Smoking	Negative	29	20.0	116	80.0	145	100.0	1*
	Positive	1	20.0	4	80.0	5	100.0	
Breast feeding	< 6 months	8	16.0	42	84.0	50	100.0	0.386
	≥ 6 months	22	22.0	78	78.0	100	100.0	
BMI (Kg/m^2^)	< 25	0	0.0	18	100.0	18	100.0	0.048
	25–29	14	26.9	38	73.1	52	100.0	
	≥ 30	16	20.0	64	80.0	80	100.0	

*By Fisher’s exact test

*OCP* oral contraceptive pill, *BMI* body mass index, *CA* carcinoma

Nearly one-third (30%) of the patients had serous cystadenoma, 18.7% had dermoid, and 12% had endometrioma (Table 4).

**Table 4:** Distribution of patients by histopathological diagnosis

Histopathological diagnosis	Number	Percent
Serous cystadenoma	45	30.0
Dermoid	28	18.7
Endometrioma	18	12.0
Mucinous cystadenoma	16	10.7
Serous cystadenocarcinoma	14	9.3
Endometrioid adenocarcinoma	8	5.3
Hemorrhagic luteal cyst	7	4.7
Follicular cyst	6	4.0
Mucinous cystadenocarcinoma	3	2.0
Krukenberg’s tumor	3	2.0
Granulosa cell cancer	2	1.3
Total	150	100.0

Approximately one-fourth of the cases (25.3%) were suspected to be malignant according to the opinion of the senior gynecologists prior to the operation, whereas 20% were proven to be malignant by histopathological examination (Table 5). Based on tumor markers, 30 and 16.7% of the masses were suspected to be malignant according to CA 125 and MIA test results, respectively.

**Table 5:** Results of gynecologist assessment, CA 125, multivariate index assay, and histopathological findings in the cases (*n* = 150)

	Malignant		Benign	
	*n*	%	*n*	%
Gynecologist’s assessment	38	25.3	112	74.7
CA 125	45	30.0	105	70.0
MIA	25	16.7	125	83.3
Histopathology results	30	20.0	120	80.0

*CA* cancer antigen, *MIA* multivariate index assay

The sensitivities of CA 125, MIA, and clinical assessment were 83.3, 70, and 70%, respectively (Table 6). When MIA and gynecologist assessment results were combined in parallel (if either or both of these tests was positive, the result of the test was considered positive), the sensitivity increased to 93.3% at the expense of specificity, which decreased to 82.5%. In general, MIA gave relatively high sensitivity (70%), specificity (96.7%), positive predictive value (84%), negative predictive value (92.8%), and total agreement (91.3%) compared with the other tests. There was no significant difference between MIA results and histopathology results (*p* = 0.267).

**Table 6:** Validity of the tests and clinical assessment compared with histopathological results

	Sensitivity %	Specificity %	PPV %	NPV %	Agreement %	P*
CA 125	83.3	83.3	55.6	95.2	83.3	0.004
MIA	70	96.7	84	92.8	91.3	0.267
Gynecologist’s assessment	70	85.8	55.3	92	82.7	0.169
MIA and/or Clinical	93.3	82.5	57.1	98	84.7	< 0.001

*By McNemar test

*CA* cancer antigen, *MIA* multivariate index assay

**Fig. 1:**
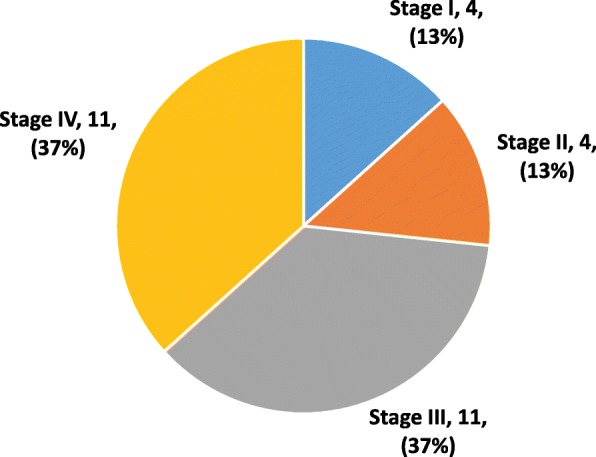
Distribution of the sample by stage of the Cancer

Out of the 150 cases sampled, 30 (19.9%) were malignant ovarian masses. Surgical staging of the 30 malignant ovarian masses determined that 13% were stage I, 13% were stage II, 37% were stage III, and 37% were stage IV (Fig. 1). The sensitivities of CA 125 and MIA in detecting stage I and stage II cancers were 75 and 100%, respectively (Table 7). The specificities were 83.3 and 96.7%, respectively. No significant difference was detected between MIA and histopathology results (*p* = 0.0125).

**Table 7:** Sensitivity of CA 125 and MIA tests in detecting stage 1 and stage 2 ovarian cancers

		Stage I & II		Benign		Total		
		*n*	%	*n*	%	*n*	%	*p*
CA 125	Positive	6	75.0	20	16.7	26	20.3	< 0.001
	Negative	2	25.0	100	83.3	102	79.7	
MIA	Positive	8	100.0	4	3.3	12	9.4	0.0125
	Negative	0	0.0	116	96.7	116	90.6	
	Total	8	100.0	120	100.0	128	100.0	

*CA 125* cancer antigen 125, *MIA* multivariate index assay

The sensitivities of CA 125 and MIA in detecting stage III and stage IV tumors were 86.4 and 59.1%, respectively (Table 8). The specificities were 83.3 and 96.7%, respectively. No significant difference was detected between MIA and histopathology results (*p* = 0.267).

**Table 8:** Sensitivity of CA 125 and MIA tests in detecting stage 3 and stage 4 ovarian cancers

		Stage III & IV		Benign		Total		
		*n*	%	*n*	%	*n*	%	
CA 125	Positive	19	86.4	20	16.7	39	27.5	< 0.001
	Negative	3	13.6	100	83.3	103	72.5	
MIA	Positive	13	59.1	4	3.3	17	12.0	0.267
	Negative	9	40.9	116	96.7	125	88.0	
	Total	22	100.0	120	100.0	142	100.0	

CA 1

## Discussion

Early detection of ovarian cancer is very important to improve patient survival. The major challenge is how to identify masses with risk of malignancy, particularly in premenopausal women [2].

In the current study, the rate of ovarian malignancy was significantly higher among postmenopausal than premenopausal women, which is consistent with a previous report [12]. Although our study showed that the rate of ovarian cancer was higher among grand multiparous women (37.5%) than nulliparous women (16.7%), parity is generally considered a well-established protective factor for ovarian cancer [13]. Bodelon et al. found that among 623 women diagnosed with invasive epithelial ovarian cancer, 102 (16%) were nulliparous and 521 (84%) were parous [14]. Tsilidis et al. found that compared with nulliparous women; parous women had a 29% lower risk of ovarian cancer with an 8% reduction in risk for each additional pregnancy [15].

The rate of malignancy was significantly higher among overweight women (BMI 25–30; 26.9%) and obese women (BMI > 30; 20%) than among women with normal BMI (0%). Beehler et al. found that compared with underweight or normal (BMI ≤ 24.9) premenopausal women, obese (BMI > 30.0) premenopausal women had an approximately 2-fold increase in the risk for malignancy. Postmenopausal women, however, did not show the same tendency, with only a small, non-significant decrease in risk among the heaviest women [16].

Our research showed no significant association between the rate of malignancy and family history of cancer (*p* = 1), oral contraceptive pill (OCP) use for more than 1 year (*p* = 0.384), fertility drugs (ovulation induction > 12 cycles) (*p* = 1), smoking (*p* = 1), and breast feeding for more than 6 months (*p* = 0.386).

A meta-analysis by Havrilesky et al. showed an inverse association between OCP use and ovarian cancer [17]. Ovarian cancer risk was 25–28% lower in women with a history of OCP use compared with never-users [18]. Risk was further reduced with longer duration of OCP use, and was decreased by more than 50% in women with more than 10 years of use [19, 20]. Ovarian cancer risk in OCP ever-users remained reduced for at least 30 years after the last use of OCPs, although the protection may diminish over time [21]. The insignificant result in our study may be related to the small number of women taking OCP.

Other studies revealed marked differences in the risk profiles of various histological types of ovarian cancer with regard to cigarette smoking [21, 22]. Current cigarette smoking increased the risk of invasive mucinous and borderline mucinous ovarian tumors, whereas former smoking increased the risk of borderline serous ovarian tumors [23]. No associations between smoking and risk of invasive serous and endometrioid ovarian cancer were observed [23].

Another study reported that breastfeeding was inversely associated with the risk of ovarian cancer; in particular, long-term breastfeeding (> 12 months) demonstrated a stronger protective effect [24].

Epithelial ovarian cancer has been described as a “silent killer”, because advanced disease is found at initial diagnosis in more than 60% of cases. In Denmark, 74% of patients were diagnosed with FIGO stage III–IV disease, compared with 60–70% in Australia, Canada, Norway, and the UK [25]. Surgical staging of ovarian tumors operated on in a tertiary care hospital revealed that more than half (56%) of the patients had stage III–IV disease. On histology, papillary serous cystic adenocarcinoma was found to be the most common type (54%), followed by mucinous 22%, endometrioid (10.6%), yolk sac (2.6%), dysgerminoma (4%), and adult granulosa cell tumor (5.3%) [26].

In one study, the individual accuracies of pelvic examination and serum CA 125 in discriminating between benign and malignant pelvic masses were approximately the same (76 and 77%, respectively) [27]. A systemic review by Kristen Pepin showed that the sensitivity of CA 125 in distinguishing between benign and malignant masses ranged from 61 to 90%, while the specificity ranged from 35 to 91%. The positive predictive value of CA 125 in women with an adnexal mass was 35 to 91%, and the negative predictive value was between 67 and 90% [28]. Miller et al. showed that replacing CA 125 with the MIA increased the sensitivity (77–94%) and negative predictive value (87–93%), while decreasing specificity (35–68%) and positive predictive value (40–52%) [29]. Similar trends were noted for premenopausal women and those with early-stage disease.

Our modified MIA demonstrated higher sensitivity and lower specificity compared with physician assessment and CA 125 in detecting ovarian malignancies. Such a noticeable improvement in sensitivity translates into a high negative predictive value, which is a clinically important measure to assure physicians and patients that the risk of malignancy will be low for patients who have a negative result by MIA. In fact, the 92.5% (149/161) sensitivity of MIA itself will produce a negative predictive value of 92.9% (156/168). The addition of MIA to clinical assessment brings significant improvement in sensitivity. This is, however, at the cost of reduced specificity [7]. Longoria et al. concluded that MIA combined with clinical assessment had significantly higher sensitivity (95.3%) compared with clinical assessment alone (68.6%) or CA 125-II (62.8%) [30]. In our research, MIA gave a higher specificity and negative predictive value compared with a study conducted by Bristoe et al., in which MIA demonstrated higher sensitivity and negative predictive value for ovarian malignancy compared with clinical impression and CA125-II [31].

MIA had a higher sensitivity than CA 125 in the detection of early-stage ovarian cancer (FIGO I & II), which was consistent with the study by Ueland et al. [7]; however, this was not the case in the detection of late-stage ovarian cancers (FIGO III & IV), for which the specificity of MIA was higher than that of CA 125 [30].

This study had several limitations. One limitation was the lack of the OvaCalc software to calculate scores; however, this is counterbalanced by the major benefit that our modified method can be used in the absence of access to software, which is the case in certain clinical settings. Another limitation is the lack of gynecologic oncology specialists in our locality.

The main strength of our study is the adaptation of available resources for discriminating ovarian tumors in a clinical setting with limited resources. Another strength of the current study lies in the inclusion of all types of ovarian malignancies that are seen in general gynecology practice. Although MIA is not intended for screening or follow-up of cases of ovarian cancer, its efficacy was proven in detecting ovarian malignancy, especially in early stages, which may significantly improve early case referral to oncology surgeons and yield a better patient survival.

## Conclusions

The modified MIA applied in this study resulted in sufficient diagnostic accuracy and appears to be highly effective for the detection of ovarian malignancy. This modified MIA strategy may be a useful method for application in developing countries.
